# Evaluation of approximate comparison methods on Bloom filters for probabilistic linkage

**DOI:** 10.23889/ijpds.v4i1.1095

**Published:** 2019-05-23

**Authors:** AP Brown, SM Randall, JH Boyd, AM Ferrante

**Affiliations:** 1 Centre for Data Linkage, Curtin University, Western Australia, Perth, Australia

## Abstract

**Introduction:**

The need for increased privacy protection in data linkage has driven the development of privacy-preserving record linkage (PPRL) techniques. A popular technique using Bloom filters with cryptographic analyses, modifications, and hashing variations to optimise privacy has been the focus of much research in this area. With few applications of Bloom filters within a probabilistic framework, there is limited information on whether approximate matches between Bloom filtered fields can improve linkage quality.

**Objectives:**

In this study, we evaluate the effectiveness of three approximate comparison methods for Bloom filters within the context of the Fellegi-Sunter model of recording linkage: Sørensen–Dice coefficient, Jaccard similarity and Hamming distance.

**Methods:**

Using synthetic datasets with introduced errors to simulate datasets with a range of data quality and a large real-world administrative health dataset, the research estimated partial weight curves for converting similarity scores (for each approximate comparison method) to partial weights at both field and dataset level. Deduplication linkages were run on each dataset using these partial weight curves. This was to compare the resulting quality of the approximate comparison techniques with linkages using simple cut-off similarity values and only exact matching.

**Results:**

Linkages using approximate comparisons produced significantly better quality results than those using exact comparisons only. Field level partial weight curves for a specific dataset produced the best quality results. The Sørensen-Dice coefficient and Jaccard similarity produced the most consistent results across a spectrum of synthetic and real-world datasets.

**Conclusion:**

The use of Bloom filter similarity comparisons for probabilistic record linkage can produce linkage quality results which are comparable to Jaro-Winkler string similarities with unencrypted linkages. Probabilistic linkages using Bloom filters benefit significantly from the use of similarity comparisons, with partial weight curves producing the best results, even when not optimised for that particular dataset

## Introduction

In recent years, record linkage centres have adopted many different models and linkage methods to ensure the protection of individual privacy as part of their operational processes. With growing demand for linked data, it has been critical for record linkage centres to implement methods which protect privacy, yet maximise the benefits that can be derived from data assets. As a result, research around privacy-preserving record linkage (PPRL) methods has become a pressing area of inquiry, with much focus on the use of Bloom filters [1-7]. Much research has focussed on the security aspect of the Bloom filters, such as cryptographic analyses of encoding methods, modifications, and hashing variations [3, 7-12]. The resultant accuracy or ‘quality’ of these techniques has often been overlooked. To consider for operational use within large-scale linkage systems, accuracy must be sufficiently high [13].

A Bloom filter is a probabilistic data structure that is used to approximate the equality of two sets; these similarity comparisons are extremely useful in record linkage allowing for typographic errors and variations in spelling. Bloom filters are implemented using an array of bits. Text values are first split into elements (typically bigrams); each element is added to the Bloom filter by applying one or more hash functions to it. The results of these hash functions determine which positions in the bit array are set to one.

Typically, PPRL techniques that use Bloom filters are applied at either the field or record level. Field level Bloom filters encode each identifier into a separate Bloom filter [14]. Record linkage techniques (deterministic and probabilistic) can then be used to link records in much the same way as with unencrypted identifiers [15-17]. Record level (or composite) Bloom filters encode two or more identifiers into a single Bloom filter [5, 18]. Composite Bloom filters may be useful in certain situations where a single linkage field is desirable or even mandated [19], but handling missing values and identifiers that change over time (such as address) remain issues [20].

Probabilistic record linkage is preferred by many data linkage centres due to its proven track record of producing high quality linkage results from unencrypted identifiers [21-23]. It has been shown to produce equally good results when applied to Bloom filters [1, 15, 16]. An extension to the basic probabilistic model of record linkage allows for approximate matches between fields. An approximate match is typically assigned a ‘similarity score’. These scores are then converted into partial weights of agreement or partial disagreement weights (as distinct from full agreement or full disagreement [24, 25]). The use of partial agreement linkage models has been shown to greatly improve the linkage quality when compared to the use of exact comparisons [25-28].

There is little mention in the literature of Bloom filters being used in the context of probabilistic record linkage where the field similarity score is converted into a partial agreement weight during the calculation of a pair-wise score [1, 15, 28]. Several issues remain unclear: What is the effect of approximate matching on the linkage quality using Bloom filters? How does this quality vary as the level of error in datasets increases? How do different approximate comparison methods perform in this context? The commonly used approximate comparisons for Bloom filters include the Sørensen–Dice coefficient, Jaccard similarity and Hamming distance [4, 14]. In this paper, we evaluate the effectiveness of each of these comparisons within the approximate comparison extensions to the Fellegi-Sunter model of record linkage [24, 29].

## Methods

### Data Sources

Synthetic data was created using an amended version of the FEBRL data generator [30]. Datasets included some core identifiers for linkage: first name, middle name, last name, sex, date of birth, address, suburb, and postcode information. The population profile of the individual fields in the master dataset was based on the frequency distributions in the Western Australian population. Western Australian addresses were randomly allocated from records in the National Address File (a public dataset containing validated Australian addresses). An additional four ‘corrupted’ datasets were created by modifying the master dataset with varying levels of error (1%, 5%, 10% and 20% of fields containing errors, respectively). The number of records allocated to each individual was based on the admission/re-admission patterns found in the Western Australian hospital morbidity data collection.

Within the ‘corrupted’ datasets, the fields containing errors were restricted to those that typically use a similarity comparison during record linkage (the ‘similarity fields’): first name, middle name, surname, address and suburb. The remaining fields were untouched. In the 1% error file, 1% of the designated fields were randomly selected to have their values corrupted, through the use of typographical errors, misspellings, truncation and replacement of values. The same procedure was used to generate a 5% error file, 10% error file and 20% error file.

Real data was also used in our evaluation. An extract from the New South Wales (NSW) Emergency Department Data Collection was used to demonstrate the effectiveness of the partial agreement methods on real-world data [31]. This dataset had previously been deduplicated to a very high standard, using full identifiers, by the Centre for Health Record Linkage (CHeReL) in NSW [32]. The results of these deduplications were used as our benchmark in determining linkage quality.

### Application of Bloom filters

Privacy-preserved versions of each dataset were created using field level Bloom filters for the ‘similarity fields’. These Bloom filters were constructed using the method first described by Schnell [14]. Fields were truncated to a maximum of twelve characters and split into bigrams that were hashed 40 times into Bloom filters 512 bits in length.

### Linkage strategy

As per the Fellegi-Sunter approach, a single block, using the date of birth field value, was applied to reduce the comparison space. This field remained untouched during the corruption process and ensured full pairs completeness for our synthetic dataset linkages. The m- and u- probabilities for each linkage field within the datasets were estimated using known matches within the block. Known matches were identified using our generated key for the synthetic datasets, and the keys provided to us for the NSW administrative dataset (our ‘truth sets’). These probabilities were used for all linkages of all datasets.

For the linkage of each dataset, the corresponding m- and u- probabilities were converted into agreement and disagreement weights as follows:

$$\text{Agreement Weight}=\log>\left(\frac{m}{u}\right)$$

$$\text{Disagreement Weight}=\log\left(\frac{1-m}{1-u}\right)$$

Fields using exact comparisons used either the full agreement weight or the full disagreement weight. Fields using approximate comparisons used a value somewhere between these two weights. A missing field value on either side of the comparison resulted in a weight of zero. The weight values were summed across all fields to determine the total ‘score’ for each pairwise comparison.

All pairs above a score of zero were recorded. Using the ‘truth set’ for each dataset, the number of missed matches (false negatives) and incorrect matches (false positives) were calculated for each possible cut-off value above zero. False negatives and false positives were treated equally, the aim to minimise the sum of these misclassifications. Thus, the cut-off value with the smallest number of misclassifications was used as the best outcome (highest quality) for that linkage. Records were grouped using transitive closure (’merge’ based) grouping, with all indirect links being honoured.

### Similarity Comparators

For linkages of the privacy-preserved datasets, the Sørensen–Dice coefficient, Jaccard similarity, and Hamming distance comparators were used to compare the similarity between Bloom filtered fields. Sørensen–Dice coefficient and Jaccard similarity scores range from 0 to 1, where higher values represent greater similarity and a score of 1 represents identical values. Similarity is based on the set of bit positions set to one in each Bloom filter. Given two of these sets, A and B, similarities are calculated as follows:

$$S(A,B)=\frac{2|A\cap B|}{|A|+|B|}$$

$$J(A,B)=\frac{|A\cap B|}{|A\cup B|}$$

Hamming distance measures the difference between values. For Bloom filters, this is the number of bits that are different between each Bloom filter and resulting scores range from 0 to the length of the Bloom filter:

H(A,B)=|A⊕B|

The raw Hamming score is normalised by dividing all raw scores by the maximum raw score giving us a value between 0 and 1; lower scores represent greater similarity, and a score of 0 represents identical values.

### Modelling partial agreement

The method for modelling partial agreement required the distribution of matches and non-matches at defined similarity scores for each field in each dataset. This was achieved by performing a deduplication linkage on each dataset and recording matches and non-matches for each observed comparison. The study used the steps outlined by Winkler for estimating partial weights at specified similarity values [24]:

1. The similarity score range for all approximate comparison used is 0..1. This range was partitioned into *i*=1,…,*N* sub-intervals. We used *N*=20 resulting in sub-intervals at 0.05 increments.
2. For each field *j* and each sub-interval $\left(k_{i},k_{i+1}\right]$, the number of matches and non-matches were recorded.
3. For each sub-interval $\left(k_{i},k_{i+1}\right]$, the match to non-match ratio *τ,_i_* was calculated as the probability of a match at interval *i* divided by the probability of a non-match at that interval:
  $$\tau_{i}=\frac{P(\delta (\gamma^{j}(a,b))\in \{(k_{i},k_{i+1}]|M\})}{P(\delta (\gamma^{j}(a,b))\in \{(k_{i},k_{i+1}]|U\})}$$
  $$\tau_{i}=\frac{{\text{matches}}_{i}/\text{totalmatches}}{{\text{nonmatches}}_{i}/\text{totalnonmatches}}$$
  $$\tau_{i}=\frac{m_{i}}{u_{i}}$$
  Here *δ* is the comparator function, *γ^j^* is a comparison of the jth field, *(a,b)* is an arbitrary pair, *M* is the set of matches, and *U* the set of non-matches.
4. The ratio vector τ is then used to create the partial weight curve for the complete set of sub-intervals *i=1,…,N*, applying the normalised ratio vector to the field weight with the disagreement weight at 0 and the agreement weight at 1.

In addition to partial weight curves, we used a simple cut-off value for the field similarity score to determine where the full agreement or full disagreement is applied. Cut-off values between 0.6 and 0.95 (in 0.05 increments) were used for all similarity fields. The cut-off value with a linkage result having the lowest number of misclassified pairs (the sum of false positives and false negatives) was selected.

### Measuring linkage quality

We used the number of misclassified pairs as a measure of linkage quality. Baselines were created for each dataset by performing deduplication linkages using exact comparisons only. Deduplication linkages using Bloom filters with each of the approximate comparisons (Sørensen–Dice, Jaccard and Hamming) were then compared to the baseline to measure the difference in linkage quality. Also, deduplication linkages using the Jaro-Winkler string comparison on unencrypted identifiers were undertaken to measure differences in linkage quality arising from the use of Bloom filters (the Jaro-Winkler comparator cannot be used directly on Bloom filtered data).

## Results

### Synthetic Data

**Figure 1: Estimated field and dataset weight curves:**
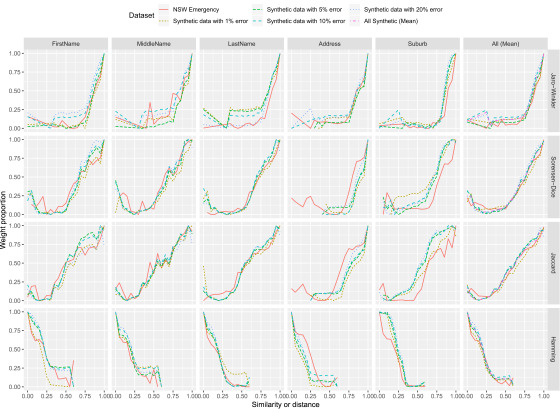
Weight proportion represents the proportion of a field match comparison weight (0 = full disagreement, 1 = full agreement)

The ‘master’ dataset of 1 million records contained multiple records belonging to the same individual. From this master dataset, a series of new datasets were created by removing or degrading the quality of particular fields. The partial weight curves were created for each field in each synthetic dataset (shown in Figure 1). Dataset level weight curves were also created as an average of the weight curves of each field; the mean of the weight proportion at each interval is used. Deduplication linkages were then performed on each of the synthetic datasets using the field level weight curves and the dataset level weight curve.

The results of the deduplication linkages for each synthetic dataset are shown in Table 1, including the linkage using ‘exact’ comparisons with field level weight curves, dataset level weight curves and the simple cut-off value that produced the fewest errors.

The performance of the similarity comparisons, when compared to the ‘exact’ comparisons, shows only a small reduction in linkage errors with the dataset containing 1% error. The benefit derived from partial agreements in data linkage appears minimal when the quality of the data is this high. However, a significant reduction in linkage error can be seen for the datasets containing at least 5% error across almost all similarity comparisons. The reduction in misclassified pairs for the dataset with 20% error, while high, is less than both the datasets with 5% and 10% error.

**Table 1: Linkage errors for each comparison (synthetic datasets):** FP = false positives, FN = false negatives, Cut-off values are shown in parentheses, Cut-off values are shown in parentheses

		1% Error			5% Error			10% Error			20% Error		

		FP	FN	Total	FP	FN	Total	FP	FN	Total	FP	FN	Total
Exact		395	1,674	2,069	2,442	18,099	20,541	131,199	16,399	147,598	110,763	372,572	483,335

Field Level													
	Jaro-Winkler	92	1,781	1,873	881	2,904	3,785	4,641	13,612	18,253	44,503	81,321	125,824
	Sørensen–Dice	125	1,713	1,838	1,054	2,517	3,571	2,978	16,736	19,714	40,436	105,024	145,460
	Jaccard	99	1,719	1,818	827	2,703	3,530	1,276	20,439	21,715	34,869	109,274	144,143
	Hamming	132	1,732	1,864	830	2,691	3,521	5,033	10,526	15,559	39,301	76,619	115,920
Dataset Level													
	Jaro-Winkler	74	1,752	1,862	1,034	2,799	3,840	3,427	15,199	17,343	47,449	84,134	135,452
	Sørensen–Dice	109	1,742	1,848	1,401	3,652	4,612	3,540	25,521	27,343	53,702	120,408	166,761
	Jaccard	83	1,744	1,819	1,205	3,708	4,563	10,691	19,047	28,179	66,948	109,909	169,002
	Hamming	72	1,753	1,871	962	2,774	3,848	3,349	13,537	16,762	29,584	101,440	129,008
Cut-off value													
	Jaro-Winkler	191	1,798	1,989	2,366	3,447	5,813	5,639	16,815	22,454	120,523	64,166	184,689
				(0.85)			(0.90)			(0.85)			(0.85)
	Sørensen–Dice	263	1,739	2,002	2,123	4,218	6,341	17,563	25,301	42,864	90,544	109,127	199,671
				(0.90)			(0.85)			(0.80)			(0.80)
	Jaccard	233	1,756	1,989	1,500	6,035	13,363	7,286	38,324	45,610	142,297	48,699	190,996
				(0.80)			(0.75)			(0.70)			(0.70)
	Hamming	155	1,806	1,961	1,710	3,677	5,387	6,799	15,687	22,486	25,428	158,455	183,883
				(0.15)			(0.15)			(0.20)			(0.20)

**Figure 2: Precision-recall for each comparison (synthetic datasets):**
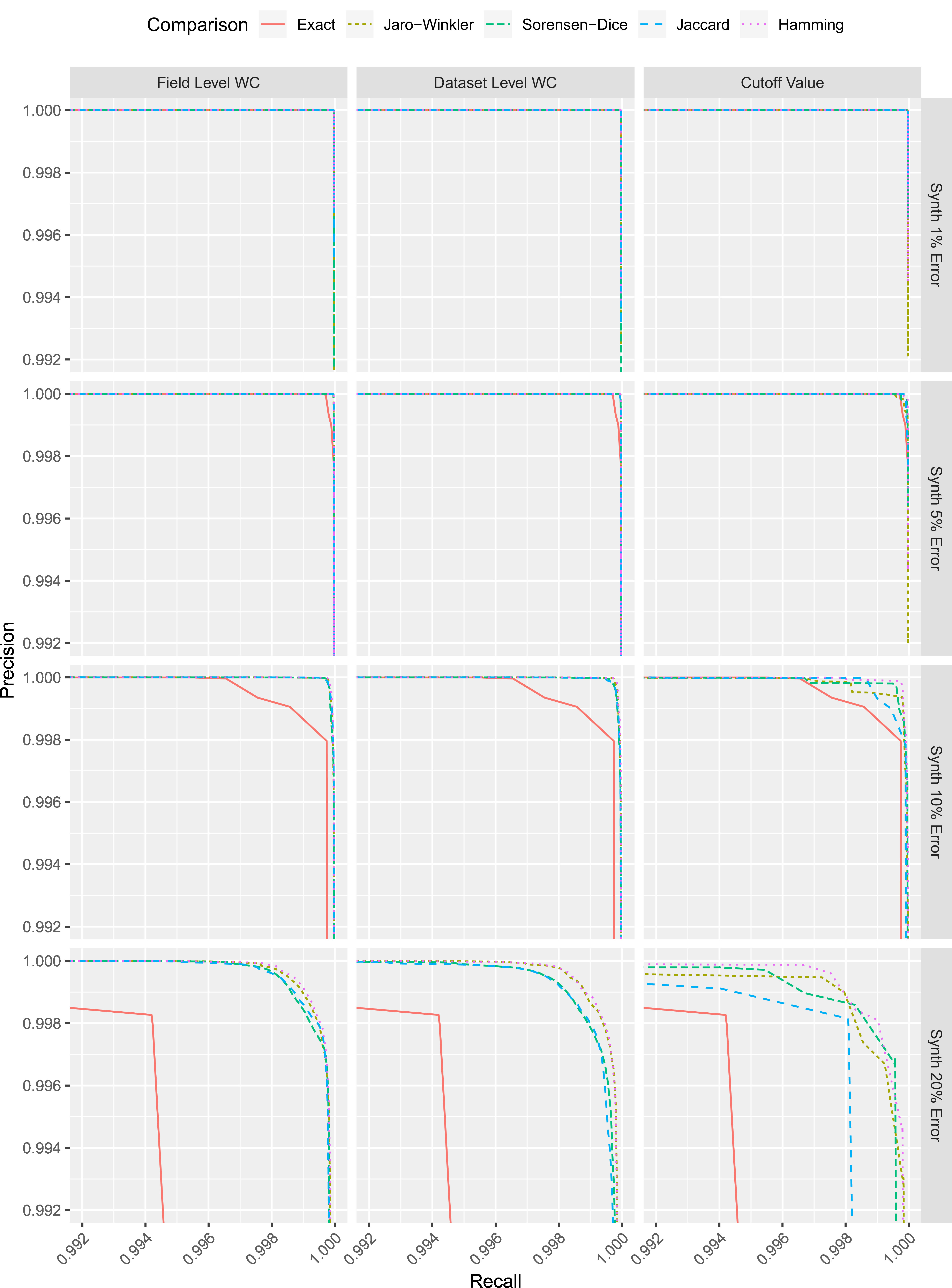
WC = weight curve

While the results in Table 1 represent the lowest error achievable for each comparison, the trade-off between precision and recall for each comparison is shown in Figure 2. When compared to exact comparisons, the field level weight curves and the dataset level weight curves for all approximate comparison methods provide a consistent improvement in recall while maintaining a high level of precision. Some instances of the comparison methods with cut-off values also provide an improvement. However, there does not appear to be the same level of consistency across all synthetic datasets.

### Evaluation on real data

The extract from the NSW Emergency Department Data Collection contained 4,304,458 records. Empty fields (missing values) were left as empty fields in the privacy-preserving version of the dataset.

Dataset level weight curves were created for the NSW Emergency dataset using the same method used with the synthetic datasets. As with the synthetic datasets, deduplication linkages were undertaken using field level, dataset level and simple cut-off values. Also, a deduplication linkage was performed using the dataset level weight curves derived from the synthetic datasets.

**Table 2: Linkage errors for each comparison (NSW Emergency dataset):** Actual cut-off values are shown in parentheses

		False Positives	False Negatives	Total

Exact		29,040	233,405	262,445
Field Level				
	Jaro-Winkler	33,729	170,188	203,917
	Sørensen–Dice	34,876	170,801	205,677
	Jaccard	44,576	162,931	207,507
	Hamming	46,905	166,138	213,043
Dataset Level				
	Jaro-Winkler	34,513	170,298	204,811
	Sørensen–Dice	41,929	176,513	218,442
	Jaccard	35,172	181,066	216,238
	Hamming	38,082	170,185	208,267
Cut-off value				
	Jaro-Winkler (0.85)	44,038	169,633	213,671
	Sørensen–Dice (0.75)	39,848	192,193	232,041
	Jaccard (0.65)	42,598	193,117	235,715
	Hamming (0.20)	39,750	191,080	230,830
Synthetic Dataset Level				
	Jaro-Winkler	31,900	172,363	204,263
	Sørensen–Dice	52,073	165,345	217,418
	Jaccard	50,586	163,769	214,355
	Hamming	40,112	170,642	210,754

**Figure 3: Precision-recall for each comparison (NSW Emergency dataset):**
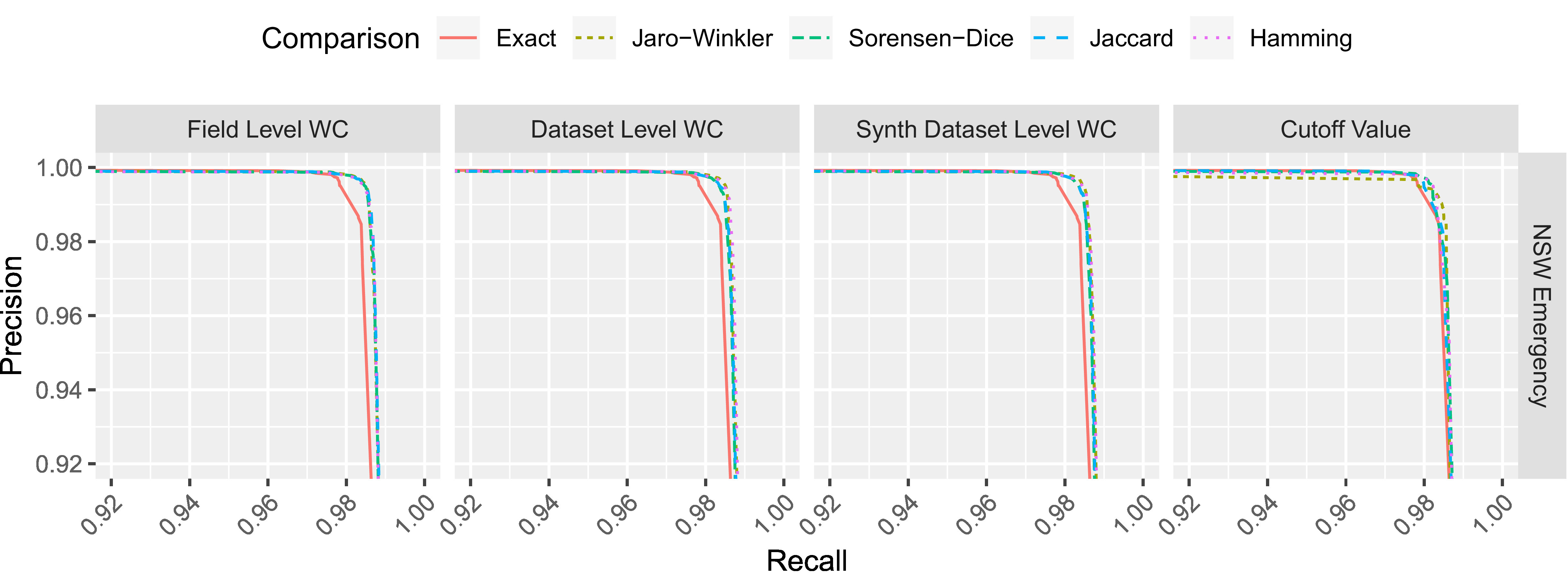
WC = weight curve

The results of all deduplication linkages on the NSW Emergency dataset are shown in Table 2, including the linkage using ‘exact’ comparisons. The trade-off between precision and recall is shown in Figure 3.

The field level weight curves produced the best results, followed by both dataset level weight curves and the use of simple cut-off values. Similarly to the synthetic datasets, the field level and dataset level weight curves demonstrate a consistent improvement to recall while maintaining a high level of precision.

## Discussion

Our results show that the use of Bloom filter similarity comparisons for probabilistic record linkage can produce linkage quality results comparable to the use of the Jaro-Winkler string similarity on unencrypted identifiers. With synthetic datasets, we found that the highest linkage quality was achieved using Hamming distance, producing fewer linkage errors (on the 10% error and 20% error datasets) than the Jaro-Winkler similarity on unencrypted identifiers. Regardless of the comparator used, all approximate comparisons improved the quality of the linkage, particularly as the level of error in the dataset increased. While the dataset with 20% error did not show the same proportional reduction (%) in misclassified pairs (as compared to datasets with only 5% and 10% error), the total number of misclassified pairs was vastly reduced. This ‘dip’ in reduction may be an artefact of the artificial error generation within the synthetic datasets, or it may be due to a limit on how much error can be accounted for using partial agreements.

As expected, optimised partial weight curves for each field produced the best quality results. The dataset level weight curves, estimated as a single ‘best-fit’ curve for all fields, showed a well defined slope for each of the comparators, with only a small increase in the number of linkage errors for both the synthetic datasets and the NSW Emergency data. The synthetic datasets and the NSW Emergency datasets produced similar weight curves, so it was unsurprising the dataset level weight curves created from the synthetic data produced high quality results on the NSW Emergency data. The fact that these results were close suggests that it may be possible to estimate a generic curve (for each comparator) for use in the linkage of various types of data; however, further testing using a variety of real datasets is warranted.

The Sørensen-Dice and Jaccard similarity comparators produced very similar linkage quality results across the range of datasets. The Hamming distance comparison appeared to produce the fewest errors for the synthetic datasets overall; however, its performance against the other comparators on the NSW Emergency data was inconsistent. This may be explained by Hamming’s observed improved performance under higher degrees of error with the synthetic datasets. If the NSW Emergency data has a similar error rate to the 1% error dataset, Hamming distance’s relative performance may also be similar.

Field level and dataset level weight curves for all approximate comparators demonstrated improvement to recall while maintaining a high level of precision, a highly desirable outcome in many linkage settings. There is still a trade-off between missed matches and incorrect matches, however, and care must be taken in selecting an appropriate cut-off during linkage.

A single cut-off value was shown to perform well in the context of determining agreement or disagreement in probabilistic linkage. The linkage quality using a cut-off value is lower than the linkage quality from an approximate weight curve (at least, for the Bloom filter comparisons), and the precision/recall trade-off is less desirable. However, the reduced level of error from an exact linkage is significant, and there appears to be some level of stability in the cut-off values themselves across our datasets. These results suggest that in the absence of being able to estimate a weight curve for a new dataset, whether it is due to size or complexity or time constraints, the use of a standard cut-off value is a viable alternative.

There were several potential limitations to this study. This work uses previously linked real data as a benchmark. While this linked data is of very high quality, it may not be completely accurate. Bloom filtered comparisons on this particular linked data provided comparable results to Jaro-Winkler comparisons. However, this does not imply that these linkage methods are therefore equivalent in all aspects; specifically, ensuring high linkage quality with privacy preserving methods will always be far more difficult, given the limited ability to provide quality assurance or clerical review. Additionally, the synthetic datasets with introduced (manufactured) errors may not always capture the complexity of real datasets. Testing the performance of the Bloom filter comparisons against other kinds of datasets or ‘gold standard’ datasets would be a valuable exercise. However, such datasets are not always easy to find [33].

## Conclusion

Matching quality in probabilistic linkage benefits significantly from the use of similarity comparisons, with partial weight curves producing the best results. We have shown that this remains true even when the weight curve has not been optimised for the particular dataset being linked. This finding also applies to the comparison of Bloom filters within a probabilistic framework. Although determining the partial weight curves for producing optimal linkage quality typically requires the use of a truth set, our results show that adequate quality can be achieved through the use of weight curves derived from simulated datasets.

All similarity comparisons produce significantly better results than ‘exact’ comparisons. Despite some of the challenges of working with Bloom filters and the range of comparators available, there is not a great difference between these comparators when used within a probabilistic framework. On the basis of these findings, our recommendation to linkage units is to choose the comparator that you are most comfortable with but to use a weight curve estimated for that particular comparator.

Conversion of similarity scores to partial agreement weights is a quality optimisation available for all approximate comparisons (including Bloom filters) and is an essential element to maximising the pair-wise quality with the Fellegi-Sunter model of record linkage. Further work is required to determine how generalisable this option is, by analysing the weight curves with a broader variety of real-world datasets.

## Ethics

Ethical approval for developing and refining linkage methodology was obtained from Curtin University Human Research Ethics Committee (HR 15/2010) as well as approval from New South Wales Cancer Institute Human Research Ethics Committee (HREC/10/CIPHS/37). Ethics approval included a waiver of consent based on the criteria in the national statement on ethical conduct in human research.

## Abbreviations

CHeReL	Centre for Health Record Linkage
FEBRL	Freely Extensible Biomedical Record Linkage
NSW	New South Wales
PPRL	Privacy Preserving Record Linkage
